# The SIRT3 and SIRT6 Promote Prostate Cancer Progression by Inhibiting Necroptosis-Mediated Innate Immune Response

**DOI:** 10.1155/2020/8820355

**Published:** 2020-11-17

**Authors:** Weiwei Fu, Hong Li, Haiyang Fu, Shuchao Zhao, Weiping Shi, Mengqi Sun, Yujun Li

**Affiliations:** Department of Pathology, The Affiliated Hospital of Qingdao University, Qingdao, Shandong 266003, China

## Abstract

The sirtuins (SIRTs), including seven family members, belong to class III histone deacetylase (HDAC) enzymes, which have been intensively investigated in cancers. Although the function of SIRTs in the cancer immunology is explored, SIRT-specific mechanisms regulating necroptosis-related innate immune response are not clear. In our present study, we found that both the mRNA and protein expression levels of SIRT3 and SIRT6 are significantly increased in the PCa tissues (HR, CI *P* = 3.30*E* − 03; HR, CI *P* = 2.35*E* − 08; and HR, CI *P* = 9.20*E* − 08) and were associated with patients' Gleason score and nodal metastasis. Furthermore, multivariate analysis showed that the PCa patients with higher expression levels of SIRT3 and SIRT6 had shorter overall survival (OS). Mechanistically, we found that SIRT3 and SIRT6 promote prostate cancer progress by inhibiting RIPK3-mediated necroptosis and innate immune response. Knockdown of both SIRT3 and SIRT6 not only activates TNF-induced necroptosis but also refreshes the corresponding recruitment of macrophages and neutrophils. Overall, our study identified that SIRT3 and SIRT6 are key regulators of necroptosis during prostate cancer progression.

## 1. Introduction

Prostate cancer (PCa) is the fifth leading cause of cancer-associated death worldwide. In 2018, over 1.3 million new cases and 359,000 deaths were reported [1], suggesting that it is urgent to identify novel diagnostic and prognostic biomarkers for PCa's better treatment. As a heterogeneous tumor, PCa undergoes epigenetic alterations, such as histone acetylation, to provide driving forces for its reprograming [2, 3]. Histone acetyltransferases (HAT) and deacetylases (HDACs) are central enzymes to alter protein acetylation [4].

The sirtuin (SIRT) family, consisting of seven members (SIRT1-7), is highly conserved NAD^+^-dependent class III histone deacetylases (HDACs) [5, 6]. Although the SIRT family shares a conserved catalytic core domain, they are functionally distinct due to their divergent enzymatic activity and cellular localization. Studies have found that the SIRT family members act as critical modulators in cellular metabolism [7], DNA repair [8], gene expression [9], mitochondrial biology in cancer [10], metabolic diseases [11], neurodegeneration [12], aging [13], etc. In addition, evidence suggests that the SIRTs have dual function in the cancer development [12]. Until now, the SIRT modulators such as nicotinamide, suramin, EX-527, sirtinol, and salermide have emerged as innovative anticancer strategies [14, 15]. These SIRT modulators have shown promising therapeutic effectiveness in lymphoma [16], glioma [17], melanoma [18], gastric cancer [19], and chronic myeloid leukemia [20]. Therefore, it is needed to further understand the clinical values of SIRTs in PCa. In fact, the clinical significance of SIRTs in PCa was found several years ago. A study has demonstrated that *SIRT6* was overexpressed in the PCa tissues compared with normal tissues, and its inhibition led to apoptosis and enhanced sensitivity of chemotherapeutical drugs [21]. In addition, *SIRT7* was increased and could serve as a predicative biomarker for PCa aggressiveness and chemoresistance [22].

Necroptosis is an inflammatory cell death, which is mediated by receptor-interacting serine/threonine-protein kinase 1 (RIPK1), RIPK3, and downstream initiator pseudokinase mixed lineage kinase domain-like protein (MLKL). Upon stimulation by TNFa, RIPK1 was recruited to the cytoplasmic membrane and formed a complex with several death-domain containing proteins, such as TRADD, TRAF2/5, and RIPK3. RIPK1 subsequently activates RIPK3, which is required for their substrate MLKL phosphorylation [23]. The phosphorylated MLKL then traffics to the membrane and enables membrane rupture and the release of cellular contents including damage-associated molecular patterns (DAMPs), thus leading to the induction of inflammation [24]. Within the whole process, the kinase activities of RIPK1 and RIPK3 are critical for activating necroptosis. Plenty of studies suggest that necroptosis is closely linked with autoimmune, inflammatory, neurodegenerative disease [25]. Several inhibitors of RIPK1 have been applied in clinical trials. One recent study identified a RIP1-HAT1-SIRT complex and demonstrated that targeting them is a promising strategy in the treatment and prevention of cancer [26] .

However, it is still unclear how SIRT3 and SIRT6 regulate RIPK1/RIPK3-mediated necroptosis and in turn maintain prostate cancer progress. In the present study, we analyzed the expression levels and genetic alterations of SIRTs in the PCa patients and tried to reveal the clinical significance of SIRTs in PCa progression using online databases. Our data showed that the expressions of both SIRT3 and SIRT6 are dramatically increased, which is closely linked with the overall survival of prostate cancer patients. With regard to the biology function of SIRT3/6, we found that SIRT3 and SIRT6 strongly control the necroptosis signaling pathway and in turn suppress the recruitment of innate immune cell macrophages and neutrophils.

## 2. Results

### 2.1. The Expression Level of SIRT3 and SIRT6 Is Upregulated in the PCa Patients

**Figure 1:**
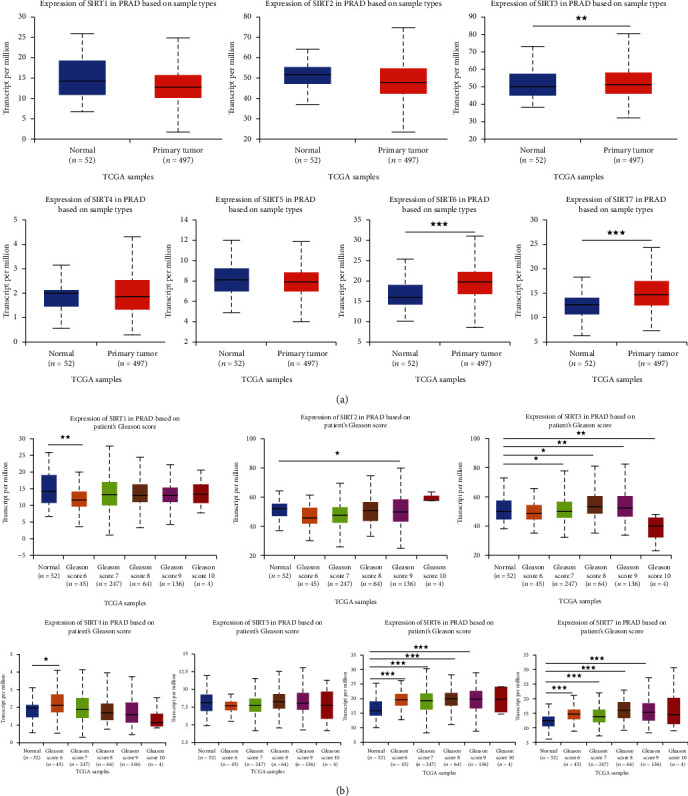
The higher expression of SIRT3 and SIRT6 in the PCa patients. (a) mRNA expression levels of SIRT3/6/7 were found to be overexpressed in primary PCa tissues compared to normal prostate samples. ^∗∗^*P* < 0.01 and ^∗∗∗^*P* < 0.001. (b) Compared to the normal prostate tissues, the mRNA expression levels of SIRT3/6/7 in PCa samples were significantly correlated with the Gleason score; SIRT1/2/4/5 did not show consistent trend. ^∗^*P* < 0.01, ^∗∗^*P* < 0.01, and ^∗∗∗^*P* < 0.001.

To explore the clinical significance of the SIRT family, we analyzed the mRNA and protein levels in the UALCAN (http://ualcan.path.uab.edu) and Human Protein Atlas websites (https://www.proteinatlas.org). As shown in Figure 1(a), higher mRNA levels of *SIRT3*, *SIRT6*, and *SIRT7* were observed in the PCa samples compared with those of the normal tissues (HR, CI *P* = 3.30*E* − 03; HR, CI *P* = 2.35*E* − 08; and HR, CI *P* = 9.20*E* − 08). However, other SIRT members indiscriminately expressed between normal and PCa tissues. The protein expressions of individual SIRT members were also examined. As indicated in Supplementary Figure 1A and 1C, the protein levels of SIRT6 and SIRT7 were obviously higher in the PCa tissues, whereas, in the normal prostate tissues, the SIRT6 and SIRT7 were rarely expressed.

**Figure 2:**
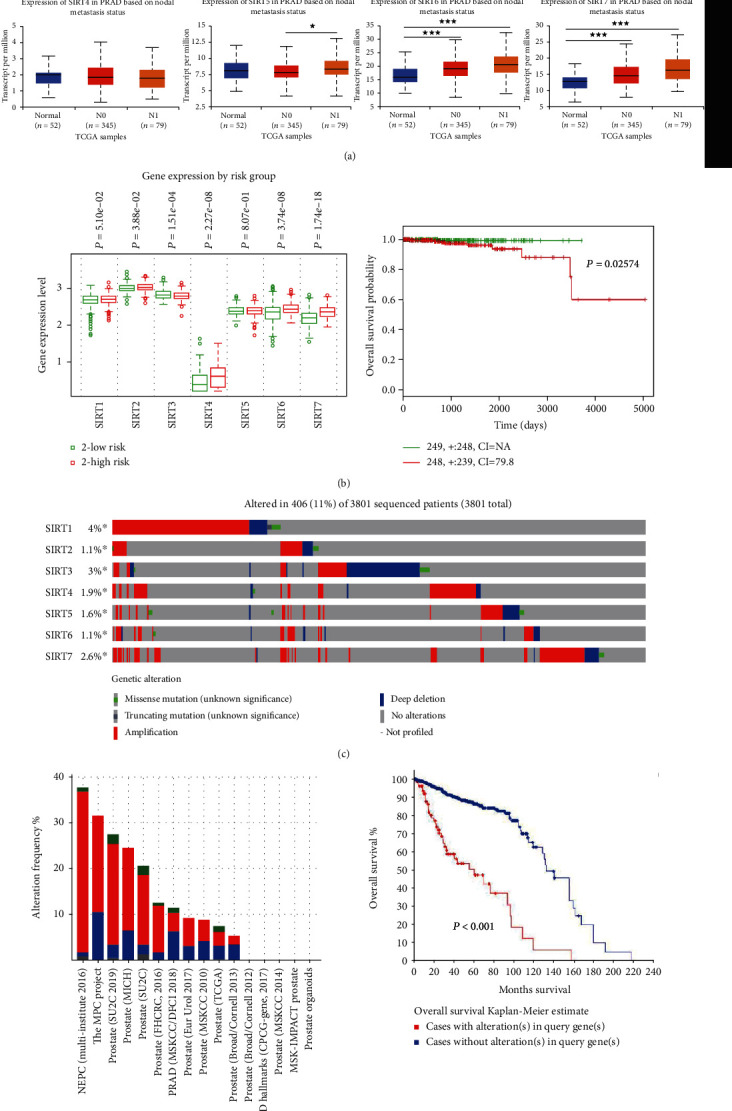
The higher expression of SIRT3 and SIRT6 in PCa patients is linked to unfavorable outcome. (a) The mRNA expression levels of SIRT3/5/6/7 were correlated with the nodal metastasis of PCa. N0: no reginal lymph node metastasis; N1: metastases in 1 to 3 axillary lymph nodes. ^∗^*P* < 0.01, ^∗∗^*P* < 0.01, and ^∗∗∗^*P* < 0.001. (b) The box plots of expression of SIRT genes in low (green) and high (red) risk groups of TCGA-PRAD patients. *x*-axis: gene expression value of each gene; *P* values are above the box plot. Kaplan-Meier survival plots showed that the high expression of the SIRTs was associated with poor survival in TCGA-PRAD patients. Red: high-risk group; green: low-risk group; top right corner inset: numbers of high- and low-risk samples, numbers of censored samples marked with and CI of each risk group; *x*-axis: time (days); *y*-axis; overall survival probability. (c) Gene alterations in SIRT genes queried from 3801 patients in 16 studies. (d) The frequency of alterations in SIRT genes in 16 individual prostate cancer studies. Genetic alterations in SIRTs were associated with shorter OS of PRAD patients.

Next, we analyzed the relationship between the mRNA expression levels of SIRT members and the clinicopathological parameters of the PCa patients in UALCAN (including patients' Gleason score and nodal metastasis status). Compared with the normal prostate tissues, the mRNA expression levels of SIRT3, SIRT6, and SIRT7 were significantly associated with the Gleason score, that is, patients with higher Gleason scores tended to have higher expression of *SIRT3*, *SIRT6*, and *SIRT7*. However, other SIRT family members did not show a consistent trend (Figure 1(b)). Importantly, patients with the nodal metastasis also tended to have higher expression levels of *SIRT3*, *SIRT6*, and *SIRT7* (Figure 2(a)), suggesting that these they may be involved in the regulation of PCa metastasis.

### 2.2. The Higher Expression of SIRT3 and SIRT6 in PCa Patients Is Linked to Unfavorable Outcome

The patients from the TCGA dataset (PCa multiforme, *n* = 499) in SurvExpress (http://bioinformatica.mty.itesm.mx:8080/Biomatec/SurvivaX.jsp) were divided into low-risk and high-risk groups using the median expression level of individual SIRT as a cutoff (Figure 2(b)). Our results showed that the high-risk PCa patients with high expression levels had poorer OS compared with the low-risk group (Figure 2(b), *HR* = 7.58, 95% CI: 82.17, and *P* = 0.026).

Then, we downloaded the clinical data (Supplementary Table 1) and mRNA expression levels of SIRTs from the FireBrowse website (http://firebrowse.org/api-docs/) for Cox survival regression analysis. In the univariate analysis, we found that age (HR = 1.039, 95% CI: 1.006-1.074, and *P* = 0.020), Gleason score (HR = 1.629, 95% CI: 1.329-1.997, and *P* < 0.001), stage (HR = 1.693, 95% CI: 1.051-2.726, and *P* = 0.030), SIRT3 (HR = 0.999, 95% CI: 0.998-1.000, and *P* = 0.014), and SIRT6 (HR = 0.999, 95% CI: 0.997-1.000, and *P* = 0.037) were all independent risk factors for OS of PCa patients (Supplementary Table 2). The multivariate analysis exhibited that PCa patients with higher mRNA levels of SIRT3 (HR = 0.998, 95% CI: 0.997-0.999, and *P* = 0.003) and SIRT6 (HR = 0.998, 95% CI: 0.997-0.999 *P* = 0.007) tended to have poorer OS (Supplementary Tables 3-9).

In addition, the results also confirmed that the SIRT6 and SIRT7 protein levels were associated with the OS of PCa patients (*P* = 0.044 and *P* = 0.017, respectively, Supplementary Figure 1B and 1D). However, other SIRT members showed no correlation with the survival (data not shown).

Genetic mutation in the SIRT gene may serve as a prognostic biomarker for PCa patients. We analyzed the genetic alterations and their associations with OS in cBioPortal (http://www.cbioportal.org). Data displayed that the predominant alteration in SIRT genes was gene amplification. And in the 3801 sequenced PCa patients, genetic alterations were observed in 183 PCa patients, and the mutation rate was 11% (Figure 2(c)). Of note, the frequency of each alteration in the SIRT gene from 16 PCa studies is shown in Figure 2(d). Furthermore, results from the Kaplan-Meier plot and log-rank test revealed that the PCa patients with genetic alterations in SIRTs tended to have shorter OS (Figure 2(d), *P* < 0.001), indicating that SIRT gene mutations indeed could affect the PCa progression.

### 2.3. Both SIRT3 and SIRT6 Control RIPK3-Induced Necroptosis

**Figure 3:**
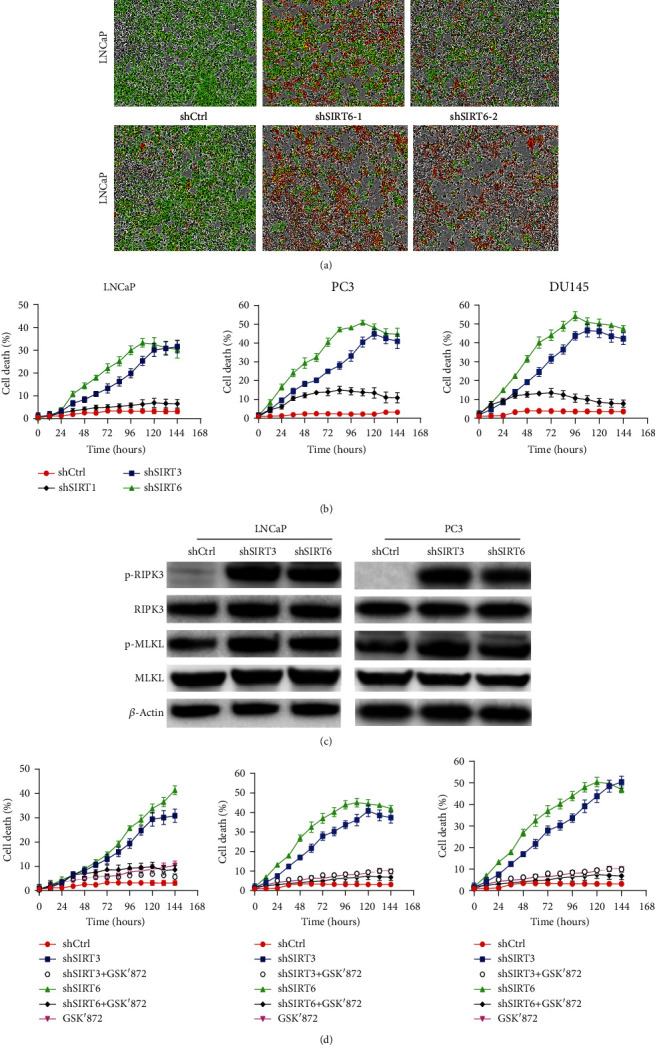
RIPK3-induced necroptosis is mediated by SIRT3 and SIRT6. (a) LNCaP cells stabilized expressed shSIRT3 and shSIRT6 with recombinant GFP and were treated with TNF (20 ng/ml). Cell death was tracked by staining with cytotoxic red and monitored by Incucyte. (b) Cell death curve of LNCaP, PC3, and DU145 cells after induction of control, SIRT1, SIRT3, or SIRT6 shRNA expression with doxycycline. (c) Western blot analysis of p-RIPK3 and p-MLKL protein levels in LNCaP and PC3 cells treated with 20 ng/ml TNF. (d) Cell death curve of LNCaP cells treated by RIPK3 inhibitor GSK′872 after induction of control, SIRT3, or SIRT6 shRNA expression with doxycycline.

Necroptosis is a kind of programmed inflammatory cell death. The dysregulated necroptosis signaling pathway is linked to various cancer progressions. A recent study showed that pan-SIRT inhibitor, MC2494, can efficiently prevent the early steps of carcinogenesis via promoting RIPK1 acetylation [26]. In order to determine whether SIRT3 and SIRT6 regulate TNF-induced necroptosis, we stably knock down either SIRT3 or SIRT6 genes that fused with a GFP cDNA controlled by an inducible promoter in prostate cell lines, LNCaP, PC3, and DU145. The cell death was analyzed by tracking a cytotoxic red signal using a live image system. As shown in Figures 3(a) and 3(b) and S2A-C, in comparing with shRNA control, loss of SIRT3 and SIRT6 dramatically increases TNF-induced cell death, which is manifested by an enhanced red signal. To validate whether shSIRT3 and shSIRT6 induce RIPK3-mediated necroptosis, we treated cells with RIPK3 inhibitor GSK′872. As shown in Figure 3(d), RIPK3 inhibition via GSK′872 completely rescues shSIRT3- and shSIRT6-induced cell death. Correspondingly, loss of SIRT3 and SIRT6 dramatically activates RIPK3 phosphorylation and their downstream effector MLKL phosphorylation (Figure 3(c)). However, there is no alteration in the expression of RIPK1 phosphorylation and cleaved caspase 8 (Figure S2B). Overall, these data suggest both SIRT3 and SIRT6 are required to control RIPK1/RIPK3-induced necroptosis.

### 2.4. Both SIRT3 and SIRT6 Promote Prostate Cancer Progress via Suppressing Necroptosis-Mediated Innate Immune Response

**Figure 4:**
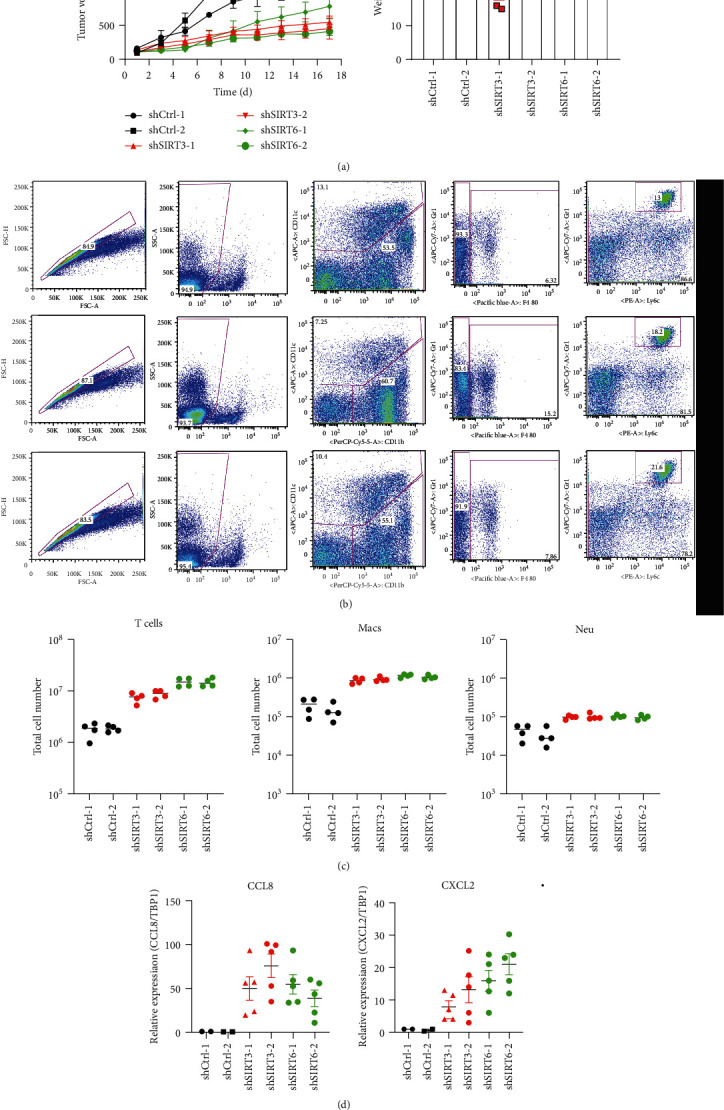
Necroptosis-induced innate immune cell recruitment controlled by both SIRT3 and SIRT6 is required to suppress prostate cancer progress. (a) Growth curves of xenografted tumors (LNCaP) after induction of control, SIRT3, or SIRT6 shRNA expression with doxycycline in vivo. Relative tumor volumes were calculated by normalizing against the tumor volume at day 1 following doxycycline administration. Body weight was tested daily. (b) Representative experiment of flow cytometric analysis of T cells, macrophages, and neutrophils in doxycycline-induced xenografted tumors (LNCaP). (c) The total number of T cells, macrophages, and neutrophils was showed in the histograms. Results represent mean ± SD of three independent experiments. ^∗^*P* < 0.05. (d) The relative expression of CCL8 and CXCL2 is shown in the histograms. GAPDH was used as the internal control. Results represent mean ± SD of three independent experiments. ^∗^P < 0.05 and ^∗∗^*P* < 0.01.

To determine whether SIRT3 and SIRT6 are required for the growth of prostate cancer in vivo, we generated the LNCaP cell line with shRNA of SIRT3 and SIRT6 after doxycycline induction using a lentivirus transduction system. These cells were subsequently injected in mice. When tumors grew to 30-60 mm^3^, shRNA expression was induced. As shown in Figure 4(a), the expression of SIRT3 and SIRT6 shRNAs dramatically inhibited tumor growth in comparison with controls. There is no alteration in the mouse body weight. Necroptosis is widely regarded as an inflammatory lytic cell death. Therefore, shSIRT3- and shSIRT6-mediated necroptosis activation would be expected to promote innate inflammation. We next assessed the recruitment of immune cells including CD4+ T cells, macrophages, and neutrophils. As shown in Figures 4(b) and 4(c), deletion of SIRT3 and SIRT6 dramatically increased the infiltration of CD4+ T cells and macrophages as well as neutrophils. Accordingly, CCL8 and CXCL2 were significantly upregulated in shSIRT3- and shSIRT6-implanted mice but showed impaired induction in shCtrl mice (Figure 4(d)). In summary, these results suggest that SIRT3 and SIRT6 promote prostate cancer progress by suppressing necroptosis-mediated immune response.

## 3. Materials and Methods

### 3.1. Ethics Statement

Our study protocol was approved by the Ethics Committee of the Affiliated Hospital of Qingdao University. As all the data were retrieved from the online databases, it could be confirmed that all informed consent had been obtained.

We utilized the UALCAN (http://ualcan.path.uab.edu) [27] which is from the TCGA database to analyze the mRNA expressions of seven SIRT members in the PCa tissues and their association with clinicopathologic parameters. Direct comparison of protein expression between human normal and cancer tissues was performed by immunohistochemistry in the Human Protein Atlas (https://www.proteinatlas.org) [28].

### 3.2. The Cancer Genome Atlas (TCGA) Database

We downloaded the PCa mRNA profile and corresponding clinical data from the TCGA database (http://gdac.broadinstitute.org/) [29]. We investigated the associations of SIRT expression with clinicopathological parameters and outcomes. The correlations between SIRT expression and clinicopathological parameters were analyzed by the chi-square (*χ*^2^) or Fisher's exact test. Statistical analyses were conducted with the software GraphPad Prism 6 and SPSS 19.0.

### 3.3. Construction and Validation of the Prognostic Gene Signature

The association of mRNA expression with survival was further analyzed with multivariate Cox regression using SurvExpress [30]. A prognosis risk score was calculated on the basis of a linear combination of seven gene expressions multiplied by a regression coefficient (*β*) derived from the multivariate Cox proportional hazard regression model of each gene with the following formula: riskscore = expressionofgene1 × *β*1gene1 + expressionofgene2 × *β*2gene2 + ⋯expressionofgene*n* × *β*7gene7. We selected the data from a total of 499 patients in the PCa cohorts available in the SurvExpress database: the TCGA-PCa cohort for individual survival analysis.

### 3.4. cBioPortal

The cBioPortal is an open access resource (http://www.cbioportal.org/) for interactive exploration of multidimensional cancer genomic data [31]. To investigate various aspects of SIRTs, genomic profiles including amplification, deep deletion, missense mutations, and copy number variance (CNV) data have been extracted from GISTIC and mRNA Expression *z*-Scores (RNASeq V2 RSEM). OS was also measured based on online instruction of cBioPortal.

### 3.5. Generation of Lentiviruses

To generate recombinant SIRT3 and SIRT6 shRNA with green fluorescent protein (GFP), we used pLKO.1 lentiviral expression vector containing the puromycin resistance gene. The lentiviruses were generated by coexpressing VSV-G and delta-8.9 in HEK-293T cells and then concentrated using PEG-it (System Biosciences).

For inducible expression of SIRT3 and SIRT6 shRNAs in tumor xenograft studies, we used pLVUTH-KRAB-KM vector with tet-inducible promotor. Cells were transduced with a lentivirus containing SIRT3 and SIRT6 shRNA for at least three days before adding puromycin for selection.

### 3.6. Western Blotting

For immunoblot analysis of necroptosis-related proteins, cell pellets were collected by trypsin digestion followed by lysis in RIPA buffer. Total protein concentration was measured with a BCA protein assay kit. Proteins were separated by electrophoresis through 4%–12% polyacrylamide gels, following electrophoretic transfer of proteins onto NC membranes with a Trans-Blot® Turbo™ Transfer System (Bio-Rad). Nonspecific binding was blocked by incubation with 5% nonfat milk, and then membranes were incubated with primary antibodies against p-RIPK3 (57220, Cell Signaling Technology [CST]), RIPK3 (95702, CST), p-MLKL (74921, CST), and MLKL (37705, CST). Membranes were then washed and incubated with the appropriate HRP-conjugated secondary antibodies (7076, anti-mouse IgG; 7074, anti-rabbit IgG). The proteins of interest were visualized by enhanced chemiluminescence (Millipore, Billerica, MA, USA).

### 3.7. RT-PCR Analysis

RNA was extracted using an RNeasy Mini Kit (74104, QIAGEN) according to the manufacturer's instructions. The isolated RNA was reverse-transcribed into cDNA using a First-Strand cDNA Synthesis Kit (4368814, Applied Biosystems). Real-time quantitative PCR was performed on an ABI 7500 RT-PCR instrument using 2x SYBR Green (4368706, Applied Biosystems) and the appropriate primers.

### 3.8. Cytotoxic Assay

LNCaP, PC3, and DU145 were cultured as described in ATCC. For cell death assay, cells were seeded in 96-well plates at 70% confluence for 1 day. On the next day, cells were stimulated with 20 ng/ml TNF (T6674, Milllipore Sigma) and 1 *μ*M GSK′872 (S8465, Selleckchem) and stained with Cytotoxic Red (4632, Essen Bioscience Inc.) following the manufacturers' protocols. The plate was scanned, and fluorescence and phase-contrast images (4 image fields/well) were acquired in real time every 2 hours after stimulation. Resulting images were analyzed using the software package supplied with the Incucyte imager (Essen Bioscience).

### 3.9. Flow Cytometry

Tumors were first dissected and then washed with ice-cold PBS. Washed tumors were cut into small pieces, which were incubated in PBS containing 10 mM HEPES, 5 mM EDTA, and 1 mM DTT at 37°C for 30 minutes with gentle shaking. The tumor segments were further digested in RPMI medium containing 0.5 mg/ml collagenase D at 37°C for 1.5 hours. The supernatant from the digested tumors was passed through a 70 *μ*m cell strainer and enriched using 37.5% Percoll to isolate immune cells. The following monoclonal antibodies were used for flow cytometry: Gr1 (RB6-8C5; 108426), F4/80 (BM8; 123116), and CD4 (GK1.5; 100408) from BioLegend; CD11b (M1/70; 48-0112-82) from Invitrogen; and CD11c (HL3; 557401) from BD Pharmingen. Cells were gated on live single-cell populations and hematopoietic cells using the CD45.2 gate followed by separation of each of the specific cell populations using the following cell surface markers: macrophages (CD11b+, F4/80+), dendritic cells (CD11c+ Gr1–), neutrophils (CD11b+, Gr1hi), and CD4 T cells (CD3+, CD4+).

## 4. Discussion

The SIRTs comprise a family of NAD^+^-dependent protein-modifying enzymes with activities in lysine deacetylation, adenosinediphospho- (ADP-) ribosylation, and/or diacylation [32]. In the present study, we intended to explore their expression levels and genetic alterations with the clinicopathological characteristics of PCa from the online public databases and assess their association with necroptosis-mediated innate immune response. Our results showed that the expression levels of SIRT3/6/7 were significantly associated with patients' Gleason score and nodal metastasis. The PCa patients with higher expression of SIRT3/6 had poorer OS, and the SIRTs' genetic alterations were served as predicative biomarkers for poor OS. Mechanistically, the higher expression of both SIRT3 and SIRT6 inhibits RIPK3 and MLKL activation, which subsequently blocks necroptosis-mediated innate immune response. Blockade of both SIRT3 and SIRT6 ameliorated necroptosis suppression. The xenograft mouse model further showed that the SIRT3 and SIRT3 blockade enhances macrophage and neutrophil recruitment and thereby suppresses prostate cancer progress. Together, all these data suggested that the SIRT expression levels and genetic mutations have essential clinical values for PCa.

*SIRT1* was widely studied in the PCa. Fu et al. [33] has found that SIRT1 interacted with androgen receptor (AR) and deacetylated its Lys^630^, leading to PCa cell growth suppression. Dai et al. [34] also found that SIRT1 acted as the AR's corepressor, and downregulation of *SIRT1* would enhance the transcriptional regulation of AR. However, Kojima et al. has reported that the upregulation of *SIRT1* could promote the PCa cell growth and induce chemoresistance in AR-negative PC3 and DU145 cells. These results suggested the dual function of *SIRT1* in PCa progression, which are dependent on the AR status. In the present study, our integrated network by cBioPortal also showed that AR was tightly related with *SIRT1*.

Until now, little was known about the roles of *SIRT2*, *SIRT3*, *SIRT4*, and *SIRT5* in the PCa. *SIRT2* was discovered as a regulator of aging in the budding yeast Saccharomyces cerevisiae [35]. Moreover, Damodaran et al. has reported that *SIRT2* deletion portended worse clinicopathological outcomes [36]. *SIRT3*, *SIRT4*, and *SIRT5* were primarily mitochondrial proteins, which have emerged as critical regulators of diverse biological events, such as cancer progression [10]. In the present study, we found that a higher expression level of *SIRT3* was associated with poorer PCa patients' OS, suggesting that it plays a critical role in the PCa development. Besides, a previous study demonstrated that *SIRT3* could suppress the PCa metastasis through promoting FOXO3A and inhibiting the Wnt/*β*-catenin pathway [37]. Inconsistent with it, our results showed that *SIRT3* was highly expressed in the PCa samples compared with the normal tissues, and its level was associated with Gleason's score and nodal metastasis. The reason is due to the androgen hormone condition, just as Lee et al. [38] showed that the expression level of *SIRT3* was overexpressed in hormone-sensitive cells (LAPC4 and LNCaP), however, reduced in castrate-resistant cells (PC3, DU145, 22RV1, and C4−2).

It is reported that the *SIRT6* was overexpressed in PCa tissues. Knockdown of *SIRT6* could lead to cell cycle arrest, apoptosis, and DNA damage through decreasing *BCL2* gene expression [21]. For *SIRT7*, Haider et al. [22] showed that its expression level was increased as PCa progressed into the high grade stage. In the present study, the upregulation of *SIRT6/SIRT7* in PCa samples and their strong association with Gleason score and nodal metastasis could confirm their oncogenic roles.

Moreover, in search of SIRT gene mutations and their 50 frequently altered neighbor genes, we found that the SIRT mutations may regulate a large number of proteins which are involved in the tumor growth. *AR*, *RB1*, and *P53*, which were implicated in the PCa onset and progression [39, 40], were highly associated with SIRT mutations. Interestingly, pathway analysis in GO and KEGG also indicated that cellular pathways, including NAD^+^-dependent histone deacetylase activity and the apoptotic signaling pathway in response to DNA damage by the p53 class mediator and regulation of cellular response to heat, were highly related to SIRT gene alterations. These findings unveiled several excellent candidates for future study.

Necroptosis has already been intensively studied in the last few decades. It was widely accepted that necroptosis is a lytic inflammatory cell death that functions as an executioner in the antitumor immunity of cancer therapy. However, the specific function of necroptosis in cancer is mysterious. One recent study demonstrated that sirtuins are able to control RIPK1-caspase 8-induced apoptosis in cancer. This occurs via a precise regulation of RIPK1 acetylation [26]. Our data corroborate the close link between SIRT3 and SIRT6 with necroptosis key adaptor RIPK3 activation. In conclusion, our study revealed the comprehensive clinical significance of SIRTs in PCa and provided clear further insights.

## Data Availability

The data used to support the findings of this study are available from the corresponding author upon request.

## Supplementary Materials

Supplementary Materials Supplementary Figure 1: representative immunohistochemistry images and prognostic values of SIRT6/7 in PCa derived from the Human Protein Atlas. (a, c) A little protein level of SIRT6 was found in normal prostate tissues, while its level was strongly detected in PCa samples. SIRT7 was expressed in PCa but not in normal prostate tissues. (b, d) Higher expression levels of SIRT6 and SIRT7 were associated with poorer OS of PCa patients. Supplementary Figure 2: RIPK3-induced necroptosis is mediated by SIRT3 and SIRT6. (a) Western blot analysis of SIRT3 and SIRT6 protein levels in LNCaP cells after induction of control, SIRT3, or SIRT6 shRNA expression with doxycycline. (b) Western blot analysis of p-RIPK1 and cleaved caspase 8 in LNCaP and PC3 cells treated with 20 ng/ml TNF. (c) PC3 cells stabilized expressed shSIRT3 and shSIRT6 with recombinant GFP and were treated with TNF (20 ng/ml). Cell death was tracked by staining with cytotoxic red and monitored by Incucyte. Supplementary Table 1-8: basic characteristics of prostate cancer patients and univariate/multivariate analysis of overall survival in prostate cancer patients. Clinical data of prostate cancer patients showed that SIRT3 and SIRT6 are all independent risk factors for OS of PCa patients, which are independent on age, Gleason score, tumor stage, and pathology stage. All of these data are from the FireBrowse website (http://firebrowse.org/api-docs/). Supplementary Table 1: basic characteristics of prostate cancer patients. Supplementary Table 2: univariate analysis of overall survival in prostate cancer patients. Supplementary Table 3: multivariate analysis of overall survival in prostate cancer patients. SIRT1. Supplementary Table 4: multivariate analysis of overall survival in prostate cancer patients. SIRT2. Supplementary Table 5: multivariate analysis of overall survival in prostate cancer patients. SIRT3. Supplementary Table 6: multivariate analysis of overall survival in prostate cancer patients. SIRT4. Supplementary Table 7: multivariate analysis of overall survival in prostate cancer patients. SIRT5. Supplementary Table 8: multivariate analysis of overall survival in prostate cancer patients. SIRT6. Supplementary Table 9: multivariate analysis of overall survival in prostate cancer patients. SIRT7.
