# Supplementary material for: The SIRT3 and SIRT6 Promote Prostate Cancer Progression by Inhibiting Necroptosis-Mediated Innate Immune Response
Source: J Immunol Res. 2020 Nov 17;2020:8820355. doi: 10.1155/2020/8820355 (PMC7685829; doi:10.1155/2020/8820355)
Supplement: Supplementary Materials — Supplementary Figure 1: representative immunohistochemistry images and prognostic values of SIRT6/7 in PCa derived from the Human Protein Atlas. (a, c) A little protein level of SIRT6 was found in normal prostate tissues, while its level was strongly detected in PCa samples. SIRT7 was expressed in PCa but not in normal prostate tissues. (b, d) Higher expression levels of SIRT6 and SIRT7 were associated with poorer OS of PCa patients. Supplementary Figure 2: RIPK3-induced necroptosis is mediated by SIRT3 and SIRT6. (a) Western blot analysis of SIRT3 and SIRT6 protein levels in LNCaP cells after induction of control, SIRT3, or SIRT6 shRNA expression with doxycycline. (b) Western blot analysis of p-RIPK1 and cleaved caspase 8 in LNCaP and PC3 cells treated with 20 ng/ml TNF. (c) PC3 cells stabilized expressed shSIRT3 and shSIRT6 with recombinant GFP and were treated with TNF (20 ng/ml). Cell death was tracked by staining with cytotoxic red and monitored by Incucyte. Supplementary Table 1-8: basic characteristics of prostate cancer patients and univariate/multivariate analysis of overall survival in prostate cancer patients. Clinical data of prostate cancer patients showed that SIRT3 and SIRT6 are all independent risk factors for OS of PCa patients, which are independent on age, Gleason score, tumor stage, and pathology stage. All of these data are from the FireBrowse website (http://firebrowse.org/api-docs/). Supplementary Table 1: basic characteristics of prostate cancer patients. Supplementary Table 2: univariate analysis of overall survival in prostate cancer patients. Supplementary Table 3: multivariate analysis of overall survival in prostate cancer patients. SIRT1. Supplementary Table 4: multivariate analysis of overall survival in prostate cancer patients. SIRT2. Supplementary Table 5: multivariate analysis of overall survival in prostate cancer patients. SIRT3. Supplementary Table 6: multivariate analysis of overall survival in prostate cancer patients. SIRT4. [file 8820355.f1.pdf]

Figure S1

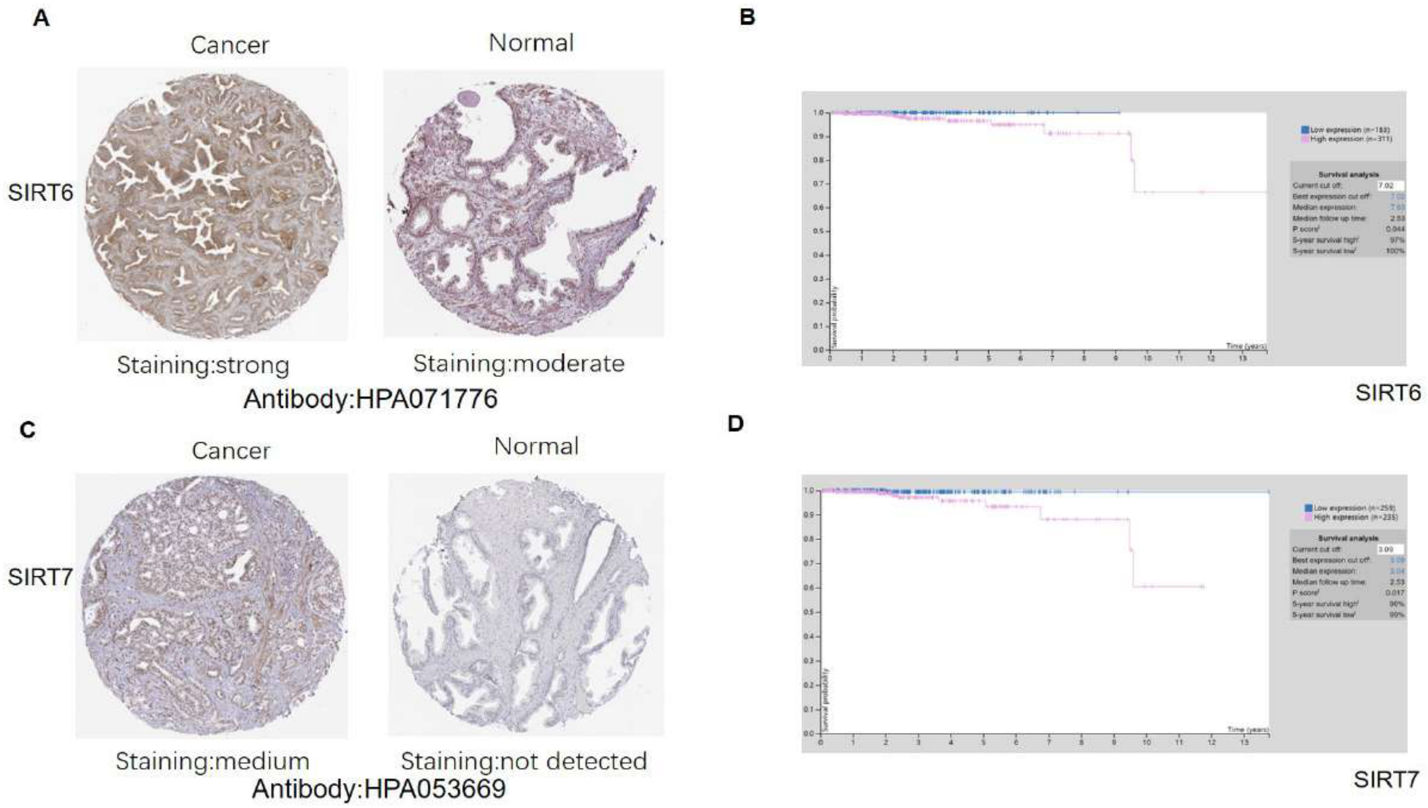

A
Cancer
Normal
SIRT6
Staining:strong
Staining:moderate
Antibody:HPA071776
B
Survival analysis
Current cut off
Best expression cut off
Median expression
Median follow up time
P score
5-year survival high
5-year survival low
SIRT6
C
Cancer
Normal
SIRT7
Staining:medium
Staining:not detected
Antibody:HPA053669
D
Survival analysis
Current cut off
Best expression cut off
Median expression
Median follow up time
P score
5-year survival high
5-year survival low
SIRT7

Figure S2

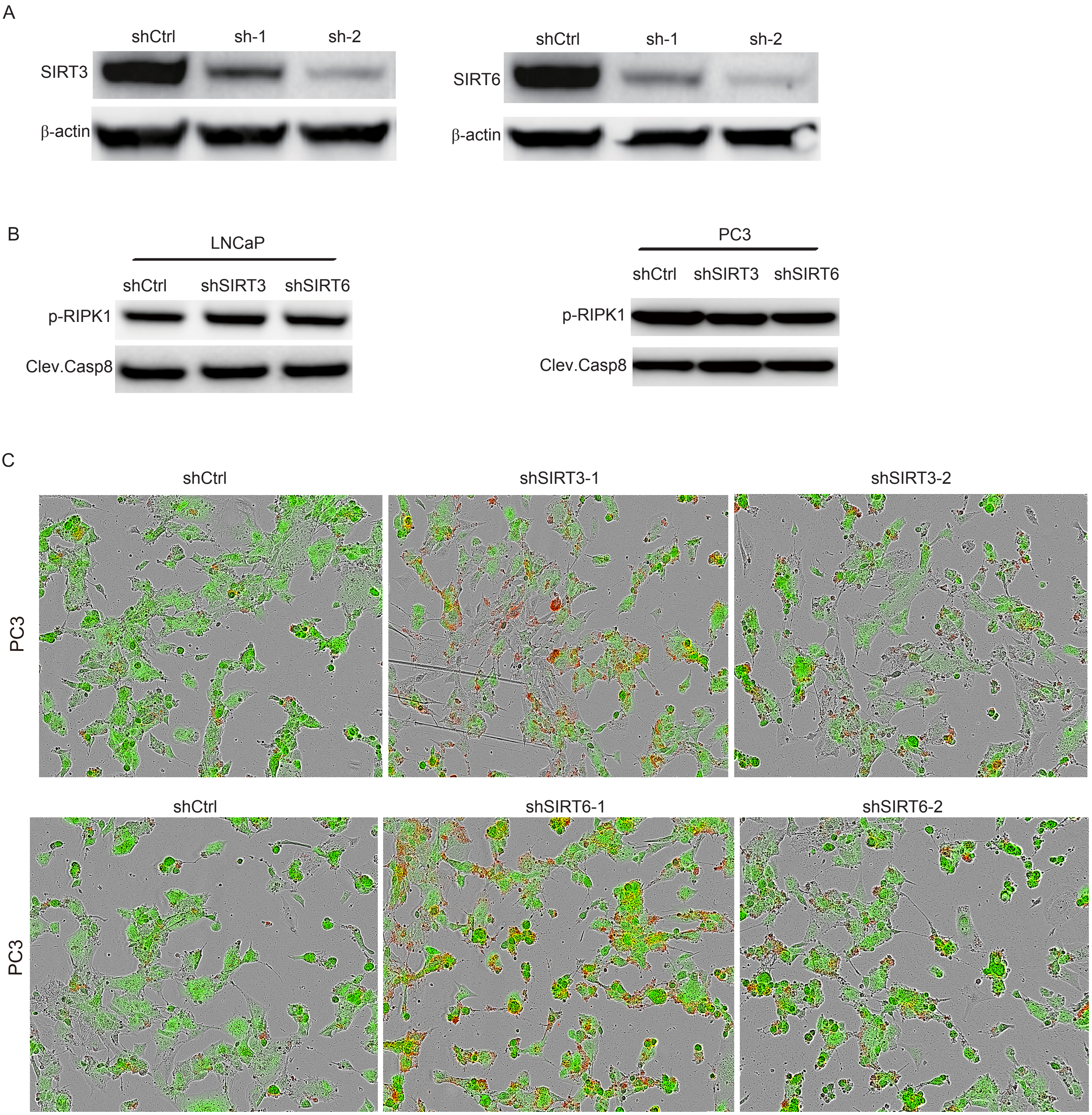
A
shCtrl
sh-1
sh-2
SIRT3
β-actin
shCtrl
sh-1
sh-2
SIRT6
β-actin
B
LNCaP
shCtrl
shSIRT3
shSIRT6
p-RIPK1
Clev.Casp8
PC3
shCtrl
shSIRT3
shSIRT6
p-RIPK1
Clev.Casp8
C
shCtrl
shSIRT3-1
shSIRT3-2
PC3
shCtrl
shSIRT6-1
shSIRT6-2
PC3

Supplementary Table 1. Basic characteristics of prostate cancer patients

Variables	PCa patients (n=499)
Age (years, Mean±SD)	61.01±6.81
Gleason scores	
<7	N=45
7	N=246
>7	N=208
Tumor stage	
≤T2b	N=23
>T2b	N=469
Pathology_N_stage	
N0	N=347
N1	N=79
PSA_value	
≥0.2	N=98
<0.2	N=344
Histological_type	
Acinar type	N=484
Other type	N=15
Amounts of gene expression (Mean±SD)	
SIRT1	663.87±217.47
SIRT2	1236.27±311.70
SIRT3	839.09±266.10
SIRT4	28.16±12.72
SIRT5	371.95±78.24
SIRT6	412.29±180.96
SIRT7	329.37±117.14

Abbreviation: PCa: prostate cancer.

Supplementary Table 2. Univariate analysis of overall survival in prostate cancer patients.

	Univariate analysis		
Variables	Hazard ratio	95% CI	*P* value
Age (years)	1.039	1.006-1.074	0.020
Gleason scores	1.629	1.329-1.997	<0.001
Tumor stage	2.675	0.657-10.889	0.170
Pathology_N_stage	1.693	1.051-2.726	0.030
Histological_type	0.538	0.196-1.474	0.228
SIRT1	1.000	0.999-1.001	0.578
SIRT2	0.999	0.999-1.000	0.158
SIRT3	0.999	0.998-1.000	0.014
SIRT4	0.999	0.982-1.015	0.869
SIRT5	0.998	0.995-1.001	0.156
SIRT6	0.999	0.997-1.000	0.037
SIRT7	1.000	0.998-1.002	0.972

Supplementary Table 3. Multivariate analysis of overall survival in prostate cancer patients.

	Multivariate analysis		
**Variables**	Hazard ratio	95% CI	*P* value
Age (years)	1.023	0.990-1.056	0.182
Gleason scores	1.437	1.140-1.810	0.002
Tumor stage	1.513	0.364-6.296	0.569
Pathology_N_stage	1.260	0.757-2.095	0.374
Histological_type	0.627	0.193-2.034	0.627
SIRT1	1.001	0.999-1.002	0.325

Supplementary Table 4. Multivariate analysis of overall survival in prostate cancer patients.

	Multivariate analysis		
Variables	Hazard ratio	95% CI	*P* value
Age (years)	1.022	0.989-1.057	0.184
Gleason scores	1.433	1.137-1.807	0.002
Tumor stage	1.485	0.358-6.158	0.586
Pathology_N_stage	1.214	0.728-2.025	0.458
Histological_type	0.509	0.149-1.737	0.281
SIRT2	0.999	0.999-1.000	0.180

Supplementary Table 5. Multivariate analysis of overall survival in prostate cancer patients.

	Multivariate analysis		
Variables	Hazard ratio	95% CI	*P* value
Age (years)	1.020	0.988-1.053	0.227
Gleason scores	1.433	1.138-1.806	0.002
Tumor stage	1.376	0.332-5.712	0.660
Pathology_N_stage	1.263	0.761-2.097	0.367
Histological_type	0.408	0.122-1.364	0.146
SIRT3	0.998	0.997-0.999	0.003

Supplementary Table 6. Multivariate analysis of overall survival in prostate cancer patients.

	Multivariate analysis		
Variables	Hazard ratio	95% CI	*P* value
Age (years)	1.023	0.990-1.057	0.181
Gleason scores	1.441	1.145-1.815	0.002
Tumor stage	1.463	0.352-6.083	0.601
Pathology_N_stage	1.262	0.759-2.098	0.370
Histological_type	0.685	0.213-2.204	0.526
SIRT4	0.999	0.983-1.016	0.939

Supplementary Table 7. Multivariate analysis of overall survival in prostate cancer patients.

	Multivariate analysis		
Variables	Hazard ratio	95% CI	*P* value
Age (years)	1.021	0.988-1.055	0.223
Gleason scores	1.443	1.146-1.818	0.002
Tumor stage	1.465	0.353-6.078	0.599
Pathology_N_stage	1.217	0.731-2.208	0.451
Histological_type	0..599	0.183-1.957	0.396
SIRT5	0.998	0.996-1.001	0.236

Supplementary Table 8. Multivariate analysis of overall survival in prostate cancer patients.

	Multivariate analysis		
Variables	Hazard ratio	95% CI	*P* value
Age (years)	1.022	0.989-1.055	0.191
Gleason scores	1.444	1.145-1.822	0.002
Tumor stage	1.480	0.357-6.135	0.589
Pathology_N_stage	1.279	0.770-2.124	0.342
Histological_type	0.415	0.125-1.376	0.150
SIRT6	0.998	0.997-0.999	0.007

Supplementary Table 9. Multivariate analysis of overall survival in prostate cancer patients.

	Multivariate analysis		
Variables	Hazard ratio	95% CI	*P* value
Age (years)	1.023	0.990-1.057	0.169
Gleason scores	1.465	1.162-1.846	0.001
Tumor stage [a]	1.442	0.347-5.990	0.614
Pathology_N_stage	1.278	0.768-2.127	0.346
Histological_type	0.511	0.151-1.734	0.281
SIRT7	0.999	0.997-1.001	0.153
